# Muscle-in-Vein Conduits for the Treatment of Symptomatic Neuroma of Sensory Digital Nerves

**DOI:** 10.3390/jpm12091514

**Published:** 2022-09-15

**Authors:** Ines Ana Ederer, Jonas Kolbenschlag, Adrien Daigeler, Theodora Wahler

**Affiliations:** 1 Department of Hand, Plastic, Reconstructive, and Burn Surgery, BG Unfallklinik, Eberhard Karls University Tuebingen, 72076 Tuebingen, Germany; 2 Department of Plastic and Aesthetic, Reconstructive and Hand Surgery, AGAPLESION Markus Hospital, 60431 Frankfurt, Germany; 3 Department of Hand, Plastic and Aesthetic Surgery, Medius Hospital Nuertingen, 72622 Nuertingen, Germany

**Keywords:** hand surgery, neuroma, digital nerve reconstruction, muscle-in-vein conduit, secondary nerve repair, restoration of function, pain reduction

## Abstract

Background: Considering the debilitating burden of neuroma resulting in a significant loss of function and excruciating pain, the use of muscle-in-vein conduits (MVCs) for the reconstruction of painful neuroma of sensory nerves of the fingers was assessed. Methods: We retrospectively analyzed 10 patients who underwent secondary digital nerve repair by MVCs. The recovery of sensibility was evaluated by static and moving two-point discrimination (2PDs, 2PDm) and Semmes-Weinstein monofilament testing (SWM). The minimum follow-up was set 12 months after the operation. Results: The median period between trauma and nerve repair was 13.4 weeks (IQR 53.5). After neuroma resection, defects ranged from 10–35 mm (mean 17.7 mm, SD 0.75). The successful recovery of sensibility was achieved in 90% of patients after a median follow-up of 27.0 months (IQR 31.00). The mean 2PDs and 2PDm was 8.1 mm (SD 3.52) and 5.2 mm (SD 2.27), respectively. Assessment by SWM resulted in a mean value of 3.54 (SD 0.69). Reduction in pain was achieved among all patients; eight patients reported the complete relief of neuropathic pain. There was no recurrence of neuroma in any patient. Conclusions: Muscle-in-vein conduits provide an effective treatment for painful neuroma of digital nerves, resulting in satisfactory restoration of sensory function and relief of pain.

## 1. Introduction

Surgeons are frequently faced with injuries to digital nerves due to lacerations, crush injuries or sharp dissections. Abnormal sensation due to neurapraxia, which temporarily limits signal transduction and resolves spontaneously, may only be present in a small number [1]. Most cases presenting with a loss of function are related to more severe nerve injuries, which require proper surgical treatment [2]. When misdiagnosed or not sufficiently addressed, aberrant axonal sprouting can lead to neuroma formation, which may significantly impede hand function.

There are plenty of options for the surgical treatment of neuroma, though no single procedure is considered universally effective [3]. In general, a distinction must be made between the treatment of end-neuroma, commonly related to amputations, and neuroma-in-continuity. In cases of no available distal nerve stump, as often seen in end-neuroma, “passive” or “ablative” options such as intraosseous implantation or nerve capping, which do not facilitate functional recovery, have to be considered. Neuroma-in-continuity, however, may be subject to nerve reconstruction in cases of two stumps after neuroma resection [4]. In accordance with primary nerve repair, reconstructive techniques aim for the tensionless restoration of nerve continuity. In this regard, autologous nerve grafts are considered the gold standard, even though they face the potential risks of hypoesthesia and de novo formation of neuroma at another site distant from the injury. Hence, patients and surgeons might be reluctant to use such a graft, especially when a small sensory nerve is to be reconstructed. As an alternative, decellularized allografts and other techniques of tubulization with either synthetic (e.g., caprolactone, polyglycolic acid or collagen—so called “conduits”) or autologous materials (e.g., veins, skeletal muscle) are to be considered [5]. Clinical data on allografts have resulted in comparable outcomes to those obtained by autografts, but considerable costs have limited their broad use for digital nerve repair so far. This further applies to manufactured conduits, which additionally face the limited indication to “small-diameter, noncritical sensory nerves with a gap of less than 3 cm” [6]. Reports about foreign body reactions with subsequent infection or implant extrusion as well as the highest rate of incomplete sensory recovery compared to allografts or autografts (27% vs. 0 vs. 12%) explain the restricted recommendation [7].

Favoring the use of autologous tissue and limiting donor site morbidity to a minimum, muscle-in-vein conduits (MVCs) are a good compromise. As demonstrated in experimental studies, MVCs provide a favorable environment for the ingrowth of regenerating axons [8,9]. The muscle inside the vein not only prevents its collapse but also acts as a natural guidance for axons [10,11]. Thus, MVCs have been successfully translated to clinics and have led to promising results regarding the reconstruction of digital nerves [12,13,14]. Their indication for painful neuroma, however, has been poorly investigated so far. Previous studies have focused on their regenerative capacities rather than their influence on neuropathic pain. We hypothesize that MVCs may provide a sufficient sheathing of the nerve from scarring tissue, leading to satisfactory reduction of neuropathic pain while enabling sensory recovery. Thus, the aim of this study was to evaluate the clinical outcomes of patients who underwent secondary nerve repair of sensory nerves of the fingers due to painful, post-traumatic neuroma.

## 2. Materials and Methods

We retrospectively reviewed all patients who underwent secondary digital nerve reconstruction by MVCs due to the presence of neuroma from 2017. The diagnosis of neuroma was made by a thorough clinical examination based on the following symptoms: hyperalgesia to touch or movement, sensation of sharp, electrically radiating pain, a positive Tinel’s sign and loss of sensory function. In addition, all patients had a history of nerve injury or suspected nerve injury several weeks before the first appointment in our clinics. The zone of nerve injury was limited to proper digital nerves of the palm or fingers located between the metacarpophalangeal joint and the distal interphalangeal joint. In all cases, verification of the neuroma was made on surgical examination. The exclusion criteria were nerve injuries related to subtotal or total amputations and pre-existing neurological disorders (e.g., polyneuropathy, diabetes) or symptoms of nerve entrapment limiting intra-individual comparison.

In total, 12 patients met the inclusion criteria and were invited to a clinical follow-up examination at least 12 months postoperatively. Due to missing data, two patients had to be excluded, which resulted in a study population of 10 patients.

### 2.1. Surgical Treatment

**Figure 1:**
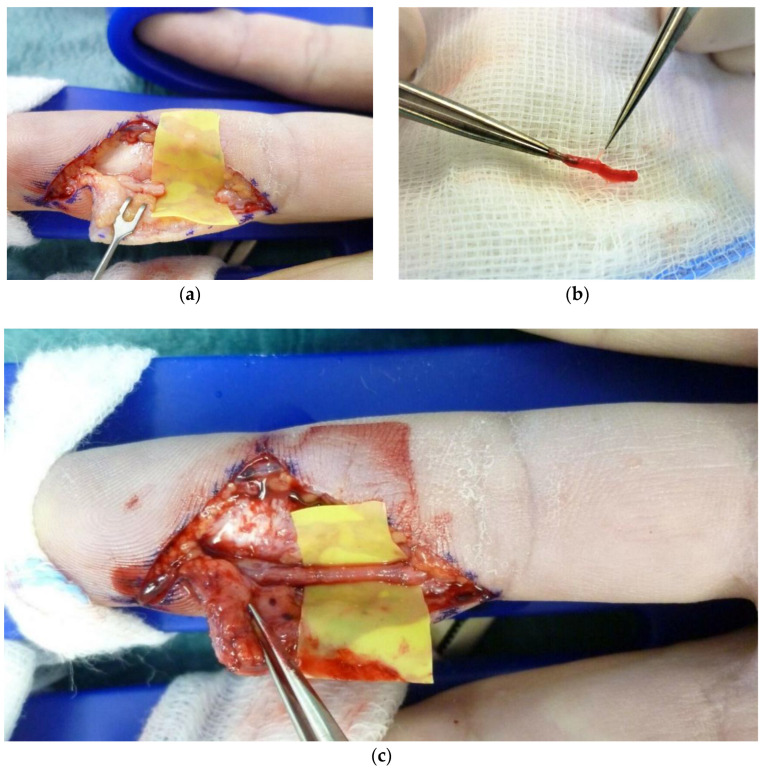
Intraoperative photographs demonstrating the reconstruction of the radial digital nerve of the ring finger by MVC. (**a**) Resection of the neuroma and measurement of the residual nerve defect. (**b**) Fashioning of the MVC by pulling the vein over the muscle fibres. (**c**) Completed nerve reconstruction with MVC in place.

After the debridement of the injured nerve, the neuroma was resected until both nerve stumps showed no residual of interfascicular scarring but normal morphological appearance under magnification (Figure 1a). The resulting nerve gap was measured with the wrist and fingers in a resting position. For reconstruction by MVCs, a subcutaneous vein and a small piece of muscle were harvested from the volar aspect of the forearm, as previously described [15]. The diameter and length of the vein were taken about 5–10 mm longer than the nerve defect and slightly wider than the nerve’s diameter to avoid the nerve’s tension and constriction, respectively. The MVC was fashioned by pulling the vein over the longitudinal course of the muscle using micro-forceps or a micro-needle holder (Figure 1b). It was then positioned between the pertaining nerve ends, while meticulous attention was given to securely overlap all fascicles (Figure 1c). Thus, the vein was pulled at least 2–3 mm over each nerve stump before being sutured epineurally, for which a non-absorbable suture of 9–0 or smaller was used.

### 2.2. Follow-Up

Clinical examination included the evaluation of sensibility and the presence of neuroma at the site of reconstruction. As previously described, Homecraft Rolyan© Semmes-Weinstein monofilaments (SWM) and a two-point discriminator (Touch-Test©, North Coast Medical Inc., Morgan Hill, CA, USA) were used for the evaluation of the detection threshold and static and moving two-point-discrimination (2PDs, 2PDm), respectively [15,16,17]. The results of the SWM-testing were categorized according to Imai (1. Normal (N) ≤ 2.83; 2. Diminished light touch (DLT) 3.22–3.61; 3. Diminished protective sensation (DPS) 3.84–4.31; 4. Loss of protective sensation (LPS) 4.56–5.88; 5. Anesthetic (A) ≥ 6.10) [18]. Outcome data pertaining to the nerve’s sensory distribution of the injured finger were compared with contralateral side, serving as intra-individual controls. Successful sensory recovery was defined as measurable two-point discrimination (2PDs ≤ 15 mm and 2PDm ≤ 10 mm).

The pre- and postoperative quantification of pain was carried out by the Numeric Pain Rating Scale (NPRS), ranging from 0, representing “no pain”, to 10, representing the “most insufferable pain”. All patients further completed the Disabilities of the Arm, Shoulder and Hand (DASH) to assess difficulties of everyday upper extremity activities [19].

**Figure 2:**
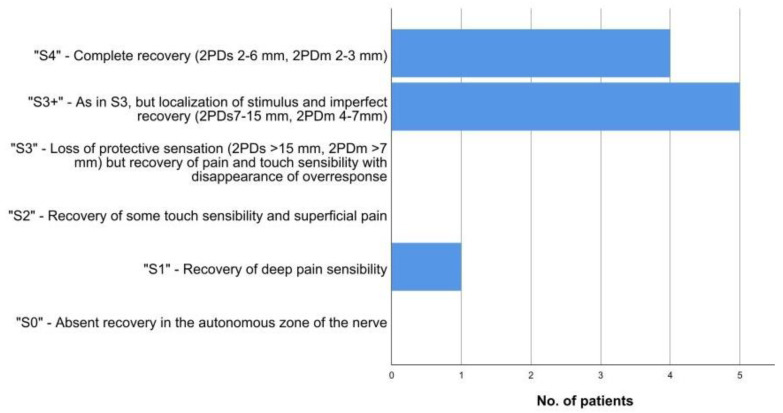
Stratification of sensory recovery by Highet and Sander’s criteria modified by Mackinnon and Dellon [21]. All but one patient regained measurable static and moving two-point discrimination (2PDs, 2PDm).

For comparison with other studies, international evaluation criteria were used, which included the modified American Society for Surgery of the Hand (ASSH) criteria and Highet and Sander’s criteria modified by Mackinnon and Dellon (Figure 2) [20,21]. According to the ASSH criteria, 2PDs was subdivided into four categories to stratify the results as follows: values of 2PDs < 6 mm were regarded as “excellent”, values of 6–10 mm were regarded as “good”, values of 11–15 mm were regarded as “fair” and values >15 mm were regarded as “poor” [20].

### 2.3. Statistical Analysis

The results are displayed as means and standard deviation (SD) or as medians and interquartile range (IQR), if appropriate. To allow for intra-individual comparison, the outcome data obtained by SWM-testing were coded ordinally (17 levels) and displayed as “level difference” between the injured and un-injured side, as previously described [15].

## 3. Results

### 3.1. Demographical Characteristics

The study comprised three females and seven males, with a mean age of 35.2 years (SD 5.5). The most common involved digital nerve was the ulnar nerve of the small finger (30%), followed by the radial nerve of the thumb, index and ring fingers—20% each, respectively. The injury mechanism mostly included sharp dissection with cutting blades or pieces of broken glass (80%). Iatrogenic nerve lesions associated with A1 pulley release were causative in two out of 10 cases. Previous nerve repair was performed in one patient whose injury was iatrogenic and immediately treated by coaptation. Among all others, nerve injuries were overseen or neglected during the initial wound assessment or wound closure, respectively. This also applied to the concomitant injuries of three patients, including two flexor tendon injuries and one dissection of the ipsilateral digital artery. Further demographic characteristics are reported in Table 1.

The median interval from injury to nerve reconstruction was 13.4 weeks (IQR 53.5, range 5.0–601.1). Following neuroma resection, nerve defects ranged from 10 to 35 mm, with a mean value of 17.7 mm (SD 0.75).

### 3.2. Evaluation of Sensibility

After a median follow-up of 27.0 months (IQR 31.00, range 12–66), meaningful sensory recovery in terms of measurable two-point discrimination was achieved in nine patients (90%). Among those, the mean 2PDs and 2PDm was 8.1 mm (SD 3.52) and 5.2 mm (SD 2.27), which correlated with a mean increase of 4.3 mm (SD 3.04) and 2.1 mm (SD 1.32) compared to the un-injured side, respectively. The results assessed by SWM-testing revealed a mean value of 3.54 (SD 0.69), which equalizes diminished light touch (DLT). Compared to the contralateral side, a mean reduction of two levels was noted after reconstruction by MVCs. Table 2 provides detailed information on follow-up evaluation.

According to the ASSH criteria, most results were regarded as excellent (20%) or good (50%), while 20% were rated as fair and 10% were rated as poor, respectively. Stratification by the modified version of Highet and Sander’s criteria (Figure 2) revealed that 90% of all results could be classified as “S4” and “S3+”, which corresponded to the aforementioned successful recovery regarding two-point-discrimination.

Respecting individual sensory perception, one patient who regained a 2PDs and 2PDm value of 4 mm and an SWM value of 2.83 reported that the sensation was normal. All others experienced subjectively different sensations at the site of reconstruction compared to the contralateral un-injured side; this even applied to three further patients who regained “S4” results according to the stratification by Highet and Sander (Patients no. 1, 3 and 5).

There were no donor site complications or operative revisions related to the reconstruction by MVCs in the observed period. One patient underwent additional surgery for the two-staged reconstruction of the flexor tendon. The recurrence of neuroma was not observed in any case.

### 3.3. Evaluation of Pain

**Figure 3:**
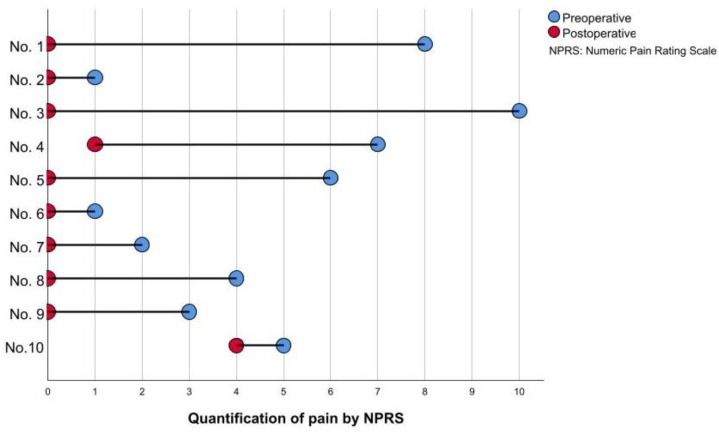
Quantification of pain NPRS (the patients´ ID is given on the *y*-axis). Comparison of pre- and postoperative values revealed an improvement by an average of 4.1 points, representing an 87% reduction from preoperative values.

The pre-operative quantification of pain revealed a mean value of 4.70 (SD 3.06). After reconstruction by MVCs, a relevant reduction could be achieved among all patients; eight patients reported the complete relief of neuropathic pain, leading to an average of 0.5 (SD 1.27) postoperatively (Figure 3).

## 4. Discussion

Personalized strategies for the operative treatment of neuroma must carefully consider anatomical characteristics as well as patients’ expectations—or, rather, considerations. The surgical treatment of neuroma is always preceded by a conscious decision on the part of the patient, wherefore the donor site morbidity must be reduced to a minimum—especially when the reconstruction of small, oligo-fascicular nerves such as digital nerves is performed. In this study, MVCs proved to be a valuable tool for the treatment of digital neuroma, enabling both the restoration of function and an adequate relief of pain. Most importantly, the recurrence of neuroma was not observed in any case, and donor site morbidity was limited to a more-or-less visible scar on the forearm which was well tolerated by all patients. Against other methods of tubulization, MVCs further allow for an individualized approach since there is abundance of autologous material, and the fashioning of the conduit can be perfectly adopted according to the nerve‘s diameter and gap length.

All but one of the patients included in this study did not undergo nerve surgery during the initial wound assessment, which was undertaken outside our department. Given the high number of neglected or misdiagnosed nerve injuries, efforts must be made to raise awareness for the proper clinical examination of hand injuries. Early diagnosis and appropriate surgical intervention are crucial to avoid the burden of neuroma formation. In this regard, we recommend performing surgical exploration in any case of suspicious nerve injury to allow for proper treatment.

Functional motor recovery significantly impairs within a few months after injury due to histopathological changes of the motor end plate and subsequent muscle fiber atrophy [22]. Sensory receptors, however, retain the potential for re-innervation, as they may survive up to several years after the injury in a kind of “atrophied” state, awaiting the arrival of an appropriate nerve terminal to allow for functional recovery [2,22]. To date, however, there is no consensus about the maximum time between injury and digital nerve repair still enabling recovery. In our study, measurable two-point discrimination was obtained 154 and 601 weeks—which are about 2.8 and 11.5 years—after injury. Similar observations were made in a recent retrospective investigation, in which 8 out of 25 neuromas were successfully repaired by processed nerve allografts more than three years following injury [23]. Two of these cases obtained meaningful recovery even more than 12 years after the initial trauma. Unfortunately, the authors did not provide detailed information on post-surgical pain, thus hindering direct comparison with our results regarding the treatment of chronic nerve injuries. Both patients of our study who underwent reconstruction several years after trauma achieved a complete relief of pain considering preoperative values of 6 and 4, evaluated by the NPRS, respectively.

Even though previous results demonstrated MVCs being comparable to autografts as far as digital nerve repair is concerned, the literature about MVCs is still scarce [15,24]. They have either fallen out of favor or been forgotten after their introduction almost three centuries ago. In 2010, a retrospective series of 21 secondary reconstructions was presented, in which neuroma-in-continuity was confirmed intraoperatively in four patients [25]. There was no further information on the remaining cases apart from the evaluation of preoperative pain, making the presence of neuroma suspicious. Additionally, the time interval between the initial trauma and nerve repair was not mentioned. Within an average follow-up of 43 months (range 18–69), complete relief of pain could be achieved among four patients, with an average postoperative value of 2 compared to 8 preoperatively. Yet, the recurrence of neuroma was recorded twice within the observed study period. Since the gap length and further demographics were comparable to those of our study, the rate of neuroma recurrence might explain the higher proportion of S3–S1 results (8/22, 36.4%) in the investigation by Marcoccio et al. In this regard, the importance of proper resecting to healthy tissue and the avoidance of tension at the site of repair must be emphasized. It is well known that tension at the site of reconstruction leads to limited intraneural perfusion, which can remarkably hinder axonal regeneration and thus increase the risk of surgical failure [26,27]. For this reason, we think the key to improving outcomes when using MVCs is to keep the segment of the vein longer than the defect, which not only avoids tension at the site of reconstruction but also allows for the overlapping of both nerve stumps to hinder aberrant axonal regeneration.

Regarding the outcome data of other reconstructive methods for the treatment of digital neuroma, our results compared favorably. In 1990, Chiu and Strauch described the successful use of autologous veins for bridging digital nerve defects following neuroma resection [28]. Even though a significant relief of pain could be obtained by this method, the recovery of sensibility was regarded as inferior compared to direct suture and autografts. Proof of effective pain reduction by vein conduits was further given by Malizos et al., who evaluated 18 patients after neuroma resection [29]. Despite these encouraging results, there are other reports indicating that functional recovery following secondary nerve repair by vein grafts may be less effective than that of primary repair, which stands in contrast to our results [30,31]. Opposed to vein conduits, MVCs can be further used for defects longer than 20–30 mm since the muscle inside the conduit hinders the vein‘s collapse [10]; thus, MVCs also serve as effective conduits for nerve regeneration in cases of longer nerve defects [14,15]. In a prospective evaluation, the use of PGA conduits for defects ranging between 5–30 mm resulted in meaningful recovery in 13 out of 15 patients (84%) [21]. The evaluation of pain was not described precisely in this study but rated by a non-standardized scale, leading to an excellent relief of pain in 40%, a good relief of pain in 33% and a poor relief of pain in 27%, respectively. As for collagen tubes, the recovery of 2PD was achieved in 10 out of 11 patients (91%) after the reconstruction of defects up to 20 mm [32]. The authors did not report on postoperative pain but evaluated the reduction of cold intolerance using a specific questionnaire, which led to normal values in about 40%. Both types of conduits did not show the recurrence of neuroma, while implant protrusion was mentioned once in the case of PGA tubes. Graft incompatibility has been a historical consideration for decellularized autografts, which have been confirmed as a safe, nonimmunogenic and successful method of reconstruction for defects up to 70 mm [33]. As for digital nerves, Taras et al. reported encouraging results, with 83% of cases showing good-to-excellent recovery and an adequate reduction in pain [34]. Considering the average interval from injury to surgery of 29 days (range 2–262) and the high incidence of concomitant injuries (7 out of 18 digits involved fractures), the indication of nerve repair is more likely to be attributable to acute nerve injuries in these cases rather than to the presence of neuroma. Another study exclusively focusing on neuroma treatment demonstrated an 80% improvement in pain and a meaningful recovery of at least S3 in 88% of all cases after allograft reconstruction [23]. However, there are also reports about undesirable outcomes including the recurrence of neuroma and the worsening of pain following the use of allografts in cases of digital neuroma [35,36].

Disregarding the varying results, patients further need to be informed that, even in the best-case scenario, sensory recovery without any subjective deficits compared to the un-injured contralateral side must be regarded an exception rather than the rule following digital nerve reconstruction [22,37]. Irrespective of the method used, careful clinical evaluation will almost always detect some sort of sensory difference. In our study, one in 10 patients reported subjectively normal sensations. After reconstruction by PGA tubes, no subjective sensory deficit was seen in two patients among a study cohort of 15 individuals [21]. Kallio et al. demonstrated that none of the 95 patients who were treated by either direct repair or fascicular grafting felt that their finger had regained normal sensation, even though meaningful recovery could be obtained in 70% of the cases [37]. Therefore, patients must be given realistic expectations, and donor site morbidity must be limited to a minimum—especially in patients suffering from neuropathic pain who fear the risk of another neuroma at a distant site from the injury. We did not observe any complications at the volar aspect of the forearm, and the scar was well tolerated by all patients. All of them further agreed on undergoing the same intervention if it was needed at another time, since convalescence was fast and positive effects in terms of pain reduction and clinical signs of recovery could be observed.

We are aware of the methodological shortcomings of this retrospective study. Owing to the small number of patients, the statistical analysis was limited to demographic description; therefore, we could not evaluate the impact of commonly discussed predictors on the surgical outcome (e.g., smoking or age). Additionally, a certain difference in follow-up must be considered, which may have had an impact on the clinical outcome. Nevertheless, our study demonstrated very well that MVCs effectively reduce neuropathic pain, with complete relief in 80% of all patients. This further contributes to the high patient satisfaction, as outlined above. As opposed to other reports on neuroma treatment, this study has eliminated the inhomogeneity of the zone of injury and the quality of the nerves included. Additionally, we provided a detailed evaluation of both functional outcome as well as neuropathic pain by comparing pre- and postoperative values. It would be imperative to now re-evaluate our results in a sufficiently powered and preferably prospective study to enable evidence-based recommendations. Finally, the efficacy of MVCs regarding neuroma treatment should be assessed in other indications, such as neuroma of the sensory branch of the radial nerve.

## 5. Conclusions

Muscle-in-vein conduits proved to be a valuable tool for the surgical treatment of painful post-traumatic neuroma-in-continuity of sensory digital nerves. The restoration of sensory function could be achieved in 90% of patients, while pain reduction was obtained among the entire cohort, with complete pain relief in 80% of all patients. Most importantly, the recurrence of neuroma was not seen in any case. Given the long regenerative potential of sensory nerves and the considerable low donor site morbidity of MVCs, this technique should also be considered for the treatment of chronic nerve injuries. Despite an extended time from injury, meaningful sensory recovery and pain relief were attainable among these patients.

## Figures and Tables

**Table 1:** Demographic patient characteristics.

Pat. ID	Age	Gender	Trauma Mechanism	Injured Nerve	Concomitant Injury	Previous Nerve Surgery	Smoking	Work-Related Injury
1	48	m	Cutter	3	No	No	No	Yes
2	31	m	Shard of glass	10	No	No	No	No
3	65	f	Iatrogenic	10	No	Coaptation	No	No
4	47	m	Knife	2	No	No	No	Yes
5	19	m	Shard of glass	10	No	No	Yes	No
6	11	m	Knife	3	Artery	No	No	No
7	29	f	Knife	1	No	No	Yes	Yes
8	54	f	Iatrogenic	7	No	No	No	No
9	26	m	Knife	1	Tendon	No	No	Yes
10	22	m	Shard of glass	7	Tendon	No	Yes	No

m: male, f: female.

**Table 2:** Postoperative results.

Pat. ID	Time Until Surgery *	Gap Length (mm)	Follow-Up (Months)	2PDs (mm)	2PDm (mm)	SWM	Imai	SWM-Level Difference **	Subjective Hypoesthesia **	DASH-Score
1	12	12	23	6	3	3.22	DLT	1	Yes	4.17
2	15	15	29	7	4	3.61	DLT	2	Yes	0.83
3	5	18	25	5	4	3.84	DPS	3	Yes	12.07
4	5	20	12	>15	>15	5.18	LPS	10	Yes	37.50
5	154	35	66	6	3	2.83	N	0	Yes	2.50
6	6	12	49	4	4	2.83	N	0	No	1.67
7	8	15	12	10	6	4.56	LPS	7	Yes	9.17
8	601	15	33	13	10	4.56	LPS	7	Yes	12.93
9	16	25	49	8	6	2.83	N	0	Yes	10.83
10	27	10	20	14	7	3.61	DLT	2	Yes	38.33

* in weeks, ** compared to contralateral side, 2PDs: static two-point-discrimination, 2PDm: moving two-point-discrimination, N: normal, DLT: diminished light touch, DPS: diminished protective sensation, LPS: loss of protective sensation.

## Data Availability

All presented data are available upon reasonable request to the corresponding author, Ederer IA. Reuse is only permitted after the agreement of all co-authors of this study.
